# Plain Capping for Improved Accuracy of Approximate One- and Two-Electron Densities at Two-Particle Coalescence Points

**DOI:** 10.1021/acs.jctc.4c00533

**Published:** 2024-09-03

**Authors:** Jerzy Cioslowski, Krzysztof Strasburger

**Affiliations:** † Institute of Physics, University of Szczecin, Wielkopolska 15, 70-451 Szczecin, Poland; ‡ Max-Planck-Institut für Physik komplexer Systeme, Nöthnitzer Straaae 38, 01187 Dresden, Germany; § Department of Physical and Quantum Chemistry, Faculty of Chemistry, Wrocław University of Science and Technology, Wybrzeże Wyspiańskiego 27, 50-370 Wrocław, Poland

## Abstract

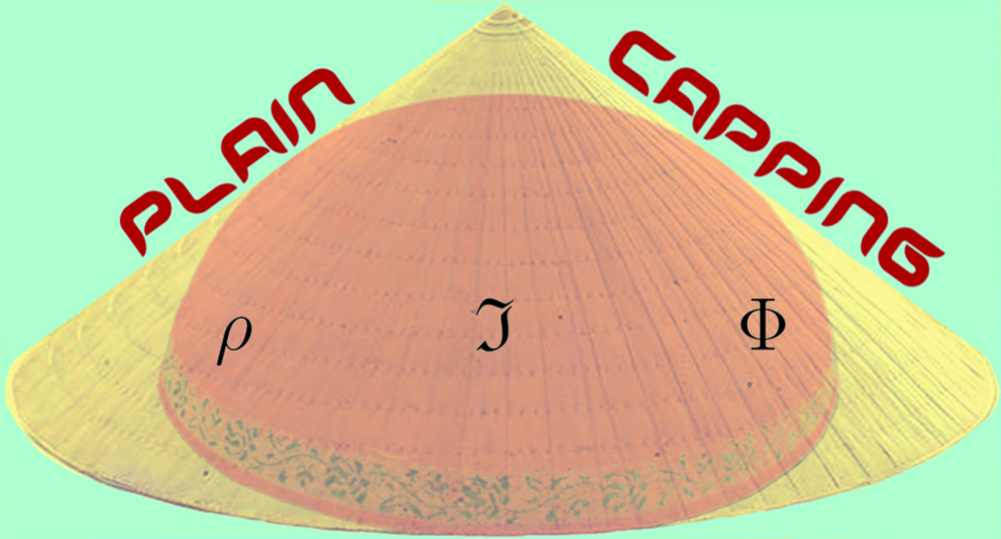

The values of the one-electron and intracule densities at two-particle coalescence points that enter the expressions for relativistic corrections to energies of Coulombic systems cannot be efficiently computed with sufficient accuracy from approximate wave functions expressed in terms of cuspless basis functions such as the explicitly correlated Gaussians. A new approach to alleviation of this problem, called plain capping, is proposed. Unlike those offered by the previously published formalisms, such as the expectation value identities and integral transforms, the accuracy improvements effected by the plain capping are attained with negligible computational effort and minimum programming. In the case of the on-top two-electron densities, whose accurate computation is particularly costly, the plain capping constitutes the only viable means of error reduction available at present.

## I Introduction

The impressive success enjoyed by modern quantum-chemical calculations in reliable predictions of electronic properties of atoms, molecules, and extended systems stems primarily from the existence of two identities, namely the product formula
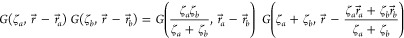
1and the integral representation
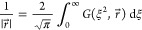
2that involve the *s*-type Gaussian primitive  whose arguments are a positive-valued exponent ζ and a three-dimensional Cartesian vector r⃗ ≡ (x,y,z). When employed in conjunction with the basis functions  of the general form
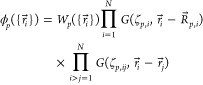
3where  comprises the position vectors , ...,  of the constituents of an N-particle system and  are polynomials in the respective vector components (*x*_1_, *y*_1_, *z*_1_), ..., (*x*_*N*_, *y*_*N*_, *z*_*N*_), these identities produce explicit expressions for the matrix elements of nonrelativistic Hamiltonians of Coulombic systems that are well suited for efficient numerical evaluation. This property of the basis functions 3, which are commonly known as the explicitly correlated Gaussians (ECGs), prompted their introduction in 1960 by Boys^1^ and Singer^2^ that followed the seminal investigation by Boys a decade earlier^3^ on the so-called Gaussian-type orbitals (GTOs), whose Hartree products correspond to the uncorrelated [i.e., those with  and ∀_*p*,*i,j*_ ζ_*p*,*ij*_ = 0] ECGs. The publication of the three landmark papers reporting on this research has heralded the commencement of the era of electronic structure calculations (usually, though not exclusively, carried out within the realm of the Born–Oppenheimer approximation) on species of interest to broad audiences of chemists and physicists.

For the relative ground-state energies of molecules as large as C_60_, the “chemical accuracy” of ∼1 [mhartree] is routinely delivered by contemporary composite methods that collate the data computed with judiciously selected combinations of diverse treatments of electron correlation and basis sets comprising various numbers and types of GTOs.^4^ Thanks to a significantly improved reproduction of the Coulomb hole at short interelectron distances, dramatic reduction of the energy error is attained upon replacement of the Hartree products of GTOs with ECGs.^5,6^ Thus, approximate electronic wave functions given by properly antisymmetrized/spin-adapted linear combinations of several thousands ECGs produce variational estimates of the nonrelativistic energies of three- and four-electron species that lie 1–10 [nhartree] above the exact values.^5,6^ However, this impressive performance does not carry over to the quantities derived from the two-electron density , such as the on-top two-electron density , the one-electron density
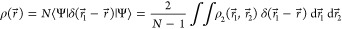
4where  is the three-dimensional Dirac delta and  is the underlying electronic wave function, and the intracule density^7,8^
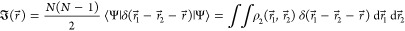
5whose values at the two-particle coalescence points [i.e., , where  is the position vector of the *I*th nucleus, for  and r⃗ = 0 for ] are predicted with far lesser accuracy. For example, one of the variational calculations quoted in the present paper (performed with 2745 ECGs) yields the nonrelativistic electronic energy of the ground state of the beryllium atom that exceeds its exact value by ca. 77 [nhartree] (which amounts to the relative error of ca. 5 × 10^–7^ %), whereas the corresponding one-electron density  at the nucleus and the intracule density  at the vanishing interelectron distance turn out to be underestimated by ca. 0.012 [bohr^–3^] (or 0.03 %) and overestimated by ca. 0.00086 [bohr^–3^] (or 0.05 %), respectively. Since these densities enter the expectation value of the Breit-Pauli Hamiltonian at the nonrecoil limit,^9,10^ the large magnitudes of these errors preclude the estimation of relativistic corrections with accuracy matching that of the computed nonrelativistic energies.

The failure of the GTO- and ECG-based electronic structure calculations to afford high-quality estimates of the one- and two-electron densities at the two-particle coalescence points stems from the presence of derivative discontinuities (known as cusps) in the electronic wave functions.^11,12^ These cusps, which persist at the one- and two-electron reduced density matrices^13,14^ and, in turn, the corresponding electron densities, cannot be reproduced with the basis functions 3 that have continuous derivatives everywhere. Consequently, the approximate one-electron and intracule densities computed with these basis functions are “too flat” in the vicinities of the two-particle coalescence points, which translates into the underestimation of the former and the overestimation of the latter.

The published approaches to the remediation of this problem follow two distinct routes, namely the introduction of the cusps in the basis functions and the reduction of the impact of cusp’s absence through sampling of the wave functions over the entire Cartesian space. Among the approaches belonging to the former category, the most radical one calls for abandoning altogether the Gaussian-type basis functions in favor of those with arguments linear rather than quadratic in the vector lengths. Although highly successful for few-electron atoms,^15^ this approach is not viable for molecules other than H_2_ (and its isoelectronic congeners) due to the very steep computational cost involved in the evaluation of the necessary integrals. A compromise solution, which has been considered thus far only for uncorrelated basis sets, calls for augmenting the GTOs with cusp-possessing one-electron auxiliaries such as the so-called Slater-type orbitals (STOs)^16^ and short-range “ramp functions”.^17,18^ Although such an augmentation does not produce excessively complicated integrals, it has the disadvantages of introducing additional *ad hoc* one-electron basis functions (and thus contributing to the undesirable proliferation of unstandardized basis sets) and of not dealing with the electron–electron coalescence cusp. These disadvantages are shared by alternative formalisms^19,20^ that, borrowing from the transcorrelated method of Boys and Handy,^21,22^ aim at removal of singularities from the nuclear attraction term in the electronic Hamiltonian.

The approaches belonging the second category refrain from altering the approximate wave functions. Instead, they rely on the expectation value identities (EVIs) that replace the Dirac deltas in eqs 4 and 5 with equivalent operators whose expectation values sample the entire Cartesian space.^5^ Implicitly incorporating the aforementioned singularities, these operators are capable of producing cusped densities from cuspless basis functions.^23^ An example of such an EVI is the Hiller–Sucher–Feinberg (HSF) formula^24^
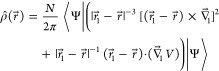
6where *V* is the potential energy that enters the electronic Hamiltonian  and  involves differentiation with respect to the components of . The failure of eq 6 to produce integrable one-electron densities from inexact wave functions^25^ is rectified in its generalizations.^23,26^ Although analogous expressions hold for the intracule density,^24,27^ the HSF formalism cannot be extended to the on-top two-electron density.

The Drachman formula^28^
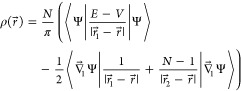
7where *E* = ⟨Ψ|*Ĥ*|Ψ⟩, is widely employed in conjunction with ECG-based calculations.^5,29^ The intracule density and several one-electron expectation values (e.g., ) have also been “drachmanized”.^29^ In principle, the on-top two-electron density can be drachmanized as well but the respective formula is not expected to be amenable to efficient evaluation. This conclusion is drawn from the comparison of the core identities, namely
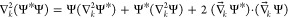
8and
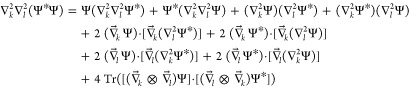
9where ⊗ stands for the direct product, from which eq 7 and its hypothetical counterpart for  respectively follow.

Yet another approach is based upon the integral representation
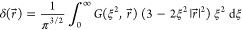
10that ensues from eq 2 upon the action of the Laplace operator. Combining eqs 4 and 10 produces the “integral-transform” (IT) expression
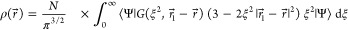
11which is evaluated as a sum of two terms, namely that involving the analytically computed integral over the range of [0, ξ_Λ_] and the remainder estimated from an asymptotic expansion incorporating the cusp in .^29,30^ The IT expression for the intracule density has a form analogous to eq 11.

Each of the aforepresented approaches has shortcomings that limit its practical usefulness. The evaluation of the expectation values of the one- and two-body operators that enter the HSF identity 6 is very involved even for the GTO-based wave functions.^31^ The expectation values of the three-body operators that appear in the analogous expression for the intracule density make its computation prohibitively complicated for wave functions other than those describing atoms and their ions within the Hartree–Fock approximation.^27^ The drachmanization is costlier still, requiring calculation of the expectation values of three-body operators for  and those of the four-body ones for . These expectation values can be evaluated analytically only in the atomic case.^29^ In contrast, the computational complexity of the IT approach is the same for all the Coulombic systems. However, the splitting of the integral that enters eq 11 introduces a degree of arbitrariness in the choices of the cutoff ξ_Λ_, the number of terms in the asymptotic expansion, and the fitting of its coefficients. It is currently unclear how variations in these arbitrary parameters affect the computed densities.

## II Theory

In light of these shortcomings, another route to the accuracy improvement of approximate one- and two-electron densities at the two-particle coalescence points is explored in the present paper. It is based upon the premise of these densities being significantly inaccurate only in the nearest vicinities of such points,^32^ thus allowing a credible reconstruction of the cusps via enforcement of the cusp conditions and smooth stitching of the reconstructed and actual densities.

Consider the one-electron density  in the vicinity of the *I*th nucleus located at . The spherical average
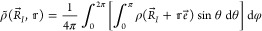
12where *e⃗* ≡ (sin θ cos φ,sin θ sin φ,cos θ) is a unit vector, conforms to the cusp condition^13^
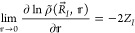
13where *Z*_*I*_ is the charge of the nucleus located at . Let
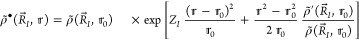
14where , be a model spherically-averaged one-electron density given by the exponential function of the lowest-degree (i.e. quadratic) polynomial of *r* that both has the proper cusp at  and produces a smooth density (of the differentiability class *C*^2^) upon stitching with . Indeed,  is compatible with the matching conditions , , and  provided the radius of stitching  satisfies the nonlinear equation
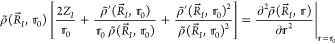
15From among the solutions of this equation, that with the smallest value is employed in actual calculations.

Replacing  with , where η(*x*) is the Heaviside step function, produces a cusp-corrected, smoothly-stitched spherically-averaged one-electron density. The estimate
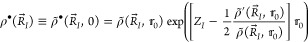
16that results from this plain capping of the one-electron density is expected to constitute an improvement over . The analogous formalism for the intracule density ensues upon replacing ρ with , *Z*_*I*_ with , and  with 0 in eqs 12–16. In the case of the on-top two-electron density, the plain capping is applied to the spherical average
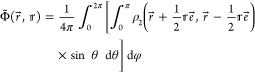
17for which an ansatz analogous to that of eq 14 is employed (with Φ̃ instead of ρ̃,  instead of *Z*_*I*_, and r⃗ instead of ). However, a detailed analysis of the near-cusp behavior of  reveals that such an ansatz is valid only for , making the applicability of the plain capping of  restricted to the position vectors r⃗ for which the radius of stitching  is not greater than . The cases of small  require a slightly more involved treatment, which will be the subject of a separate publication.

In summary, eqs 12, 15, and 16 explicitly define  as a functional of . In the cases of  and , the analogous functionals are universal (i.e. independent of the nuclear charge).

## III Numerical Examples and Discussion

Before the presentation of numerous numerical examples illustrating the desirable features of the aforedescribed formalism, a few remarks are in order. First of all, the plain capping is universally applicable to the one-electron, intracule, and on-top two-electron densities originating from reasonably accurate electronic wave functions constructed from either one-electron or explicitly correlated basis functions. Unlike the previously published approaches, it improves the accuracy of densities at two-particle coalescence points without resorting to additional basis functions or many-electron integrals, the only input quantity being the density itself. In the case of ECG-based wave functions, these densities are linear combinations of the Gaussian primitives , where the parameters {χ_*p*_} and  derive from those (i.e., {ζ_*p*,*i*_},  and {ζ_*p*,*ij*_}) entering the basis functions 3, whose spherical averages
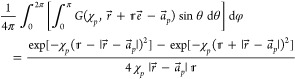
18are readily evaluated. The same holds true for their first and second partial derivatives with respect to  that follow from eq 18 via straightforward differentiation. Consequently, the computational implementation of eqs 12 and 14–18 is trivial and the computational cost of the evaluation of the quantities they involve is remarkably low. Second, the plain capping at  a positive-valued error in the normalization of  amounts to . Since the negative-valued renormalization correction to  due to this error has the magnitude of less than  that, thanks to the smallness of the prefactor , is negligible in comparison with the plain-capping correction equal to , it can be safely neglected. This conclusion carries over to the intracule and on-top two-electron densities. Third, although the ansatz 14 is somewhat reminiscent to that previously proposed for the cusp-restoring correction of molecular orbitals,^33^ the present approach employs an entirely different philosophy of being applied to densities rather than orbitals, relying on the optimum rather than fixed radius of stitching, and involving the polynomial in  with the degree set to the lowest possible value of two.

Robust testing of the efficacy of the plain capping is provided by test calculations involving approximate electronic wave functions given by the expansions , where the operator  assures the inclusion of the spin degrees of freedom and a proper permutational antisymmetry. The results of these test calculations, carried out for the ground states of several two-, three-, and four-electron species, are analyzed in terms of the accuracy improvements in the one-electron, intracule, and on-top two-electron densities effected by the plain capping.

In the case of the beryllium atom, the error in the one-electron density  at the nucleus is reduced by a factor of 7.5–934 depending on *K*, the analogous figures being 6.2–42 for the intracule density  at the electron–electron coalescence point (Table 1). The respective ratios of the errors in the original and plain-capped one-electron densities at nuclei (intracule densities at the electron–electron coalescence points) equal 12–49 (5.2–36) for the lithium anion (Table 2), 13–88 (11–20) for the H_2_ molecule (*R*_*HH*_ = 1.4011 [bohr]; Table 3), and 10–33 (2.4–8.9) for the H_3_^+^ cation (*R*_*HH*_ = 1.65 [bohr]; Table 4). As there are no relevant benchmark data available in the literature for the LiH molecule at the internuclear distance of 3.015 [bohr], another measure of the accuracy improvement, namely the reduction in the variability in the densities computed with different *K* brought about by the plain capping, has to be employed in this case. This reduction is 7.2-, 25-, and 13-fold for the one-electron densities at the Li and H nuclei, and the intracule density at the electron–electron coalescence point, respectively (Tables 5 and 6). For the lithium atom, the error in  is reduced by a factor of 4.9–155 (Table 6), the analogous figures for the α and β components of the one-electron density at the nucleus being 12–31 and 17–515, respectively (Table 7). Upon the application of the plain capping, the corresponding total density becomes 21–122 times more accurate, whereas the accuracy of its spin counterpart is actually *reduced* by a factor of 1.3–4.5 (Table 8). Interestingly, the data presented in Table 8 indicate that the summation/subtraction and plain-capping operations almost commute when performed upon the α and β density components. The variability with *K* in the on-top two-electron densities of the beryllium atom computed at various values of the distance  from the nucleus is significantly (i.e., by the factors of 19, 15, 5.8, 13, 4.9, and 8.1 for  = 1.0, 0.5, 0.2, 0.1, 0.05, and 0.02 [bohr], respectively) dampened by the plain capping (Table 9).

**Table 1:** One-Electron and Intracule Densities of the Ground State of the Beryllium Atom^a^

*K*	one-electron density at the Be nucleus		intracule density at the coalescence point	
	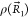	b	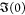	b
872	35.33429	35.36640 (3.52 × 10^–4^)	1.608407	1.605438 (5.77 × 10^–3^)
1278	35.34702	35.36608 (2.12 × 10^–4^)	1.607431	1.605356 (4.02 × 10^–3^)
1873	35.35207	35.36829 (1.79 × 10^–4^)	1.606519	1.605385 (2.23 × 10^–3^)
2745	35.35715	35.36859 (1.25 × 10^–4^)	1.606169	1.605348 (1.60 × 10^–3^)
4023	35.36127	35.36824 (7.83 × 10^–5^)	1.605809	1.605386 (8.35 × 10^–4^)
5896	35.36340	35.36900 (6.13 × 10^–5^)	1.605697	1.605329 (7.18 × 10^–4^)
lit.^c^	35.36901		1.605305	

a All densities in [bohr^–3^].

b Values of  in parentheses.

c The drachmanized values of  and  derived from an approximate electronic wave function involving 7000 ECGs.^34^

**Table 2:** One-Electron and Intracule Densities of the Ground State of the Lithium Anion^a^

*K*	one-electron density at the Li nucleus		intracule density at the coalescence point	
	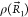	b	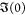	b
872	13.81914	13.83718 (6.77 × 10^–4^)	0.5453310	0.5444280 (5.11 × 10^–3^)
1278	13.82628	13.83710 (4.09 × 10^–4^)	0.5450958	0.5442389 (4.91 × 10^–3^)
1873	13.82962	13.83769 (3.02 × 10^–4^)	0.5448362	0.5442474 (3.39 × 10^–3^)
2745	13.83200	13.83785 (2.19 × 10^–4^)	0.5446668	0.5442338 (2.50 × 10^–3^)
4023	13.83439	13.83794 (1.33 × 10^–4^)	0.5444483	0.5442298 (1.26 × 10^–3^)
5896	13.83512	13.83802 (1.10 × 10^–4^)	0.5444094	0.5442238 (1.07 × 10^–3^)
lit.^c^	13.83808		0.5442142	

a All densities in [bohr^–3^].

b Values of  in parentheses.

c The drachmanized values of  and  derived from an approximate electronic wave function involving 4200 ECGs.^35^

**Table 3:** One-Electron and Intracule Densities of the Ground State of the H_2_ Molecule^a^

*K*	one-electron density at the H nucleus		intracule density at the coalescence point	
	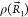	b	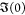	b
200	0.4588326	0.4593300 (1.68 × 10^–3^)	0.01674608	0.01671836 (5.10 × 10^–3^)
905	0.4592528	0.4593271 (2.48 × 10^–4^)	0.01672766	0.01671948 (1.50 × 10^–3^)
2676	0.4593105	0.4593233 (4.29 × 10^–5^)	0.01672071	0.01672015 (1.02 × 10^–4^)
3264	0.4593152	0.4593228 (2.57 × 10^–5^)	0.01672053	0.01672016 (6.81 × 10^–5^)
4788	0.4593187	0.4593232 (1.50 × 10^–5^)	0.01672027	0.01672018 (1.59 × 10^–5^)
lit.^c^	0.4593229		0.01672018	

a = 1.4011 [bohr]; all densities in [bohr^–3^].

b Values of  in parentheses.

c The drachmanized (and subsequently extrapolated to the complete-basis-set limit) values of  and  derived from an approximate electronic wave function involving 1024 ECGs.^36^

**Table 4:** One-Electron and Intracule Densities of the Ground State of the H_3_^+^ Cation^a^

*K*	one-electron density at the H nucleus		intracule density at the coalescence point	
	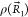	b	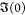	b
872	0.3631726	0.3632196 (1.98 × 10^–4^)	0.01833760	0.01833446 (5.25 × 10^–4^)
1278	0.3631866	0.3632210 (1.45 × 10^–4^)	0.01833635	0.01833456 (2.98 × 10^–4^)
1873	0.3632054	0.3632187 (5.65 × 10^–5^)	0.01833536	0.01833465 (1.18 × 10^–4^)
2740	0.3632098	0.3632189 (3.85 × 10^–5^)	0.01833508	0.01833467 (6.91 × 10^–5^)
3943	0.3632104	0.3632190 (3.61 × 10^–5^)	0.01833503	0.01833467 (5.99 × 10^–5^)
lit.^c^	0.3632182		0.01833477	

a = 1.65 [bohr]; all densities in [bohr^–3^].

b Values of  in parentheses.

c The IT-regularized values of  and  derived from an approximate electronic wave function involving 600 ECGs.^30^

**Table 5:** One-Electron Density of the Ground State of the LiH Molecule^a^

*K*	one-electron density at the Li nucleus		one-electron density at the H nucleus	
	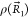	b	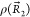	b
595	13.76679	13.81001 (1.64 × 10^–3^)	0.3812052	0.3846462 (1.40 × 10^–2^)
872	13.77767	13.81431 (1.38 × 10^–3^)	0.3824581	0.3846596 (8.94 × 10^–3^)
1278	13.79321	13.81447 (8.04 × 10^–4^)	0.3832053	0.3847357 (6.18 × 10^–3^)
1873	13.79935	13.81547 (6.08 × 10^–4^)	0.3838325	0.3847350 (3.68 × 10^–3^)
2745	13.80638	13.81546 (3.45 × 10^–4^)	0.3841103	0.3847640 (2.63 × 10^–3^)
4023	13.80775	13.81615 (3.17 × 10^–4^)	0.3843277	0.3847576 (1.77 × 10^–3^)
5896	13.81122	13.81619 (1.87 × 10^–4^)	0.3844782	0.3847763 (1.20 × 10^–3^)

a = 3.015 [bohr]; all densities in [bohr^–3^].

b Values of  in parentheses.

**Table 6:** Intracule Densities of the Ground States of the Li Atom and the LiH Molecule^a^

*K*	Li intracule density at the coalescence point		LiH intracule density at the coalescence point	
	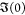	b	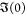	b
595	0.5449091	0.5443284 (3.34 × 10^–3^)	0.5534250	0.5492693 (2.38 × 10^–2^)
872	0.5446673	0.5443186 (1.97 × 10^–3^)	0.5516250	0.5492032 (1.39 × 10^–2^)
1278	0.5445064	0.5443273 (1.03 × 10^–3^)	0.5507963	0.5490240 (1.01 × 10^–2^)
1873	0.5444467	0.5443268 (6.89 × 10^–4^)	0.5499905	0.5490319 (5.50 × 10^–3^)
2745	0.5444076	0.5443225 (4.84 × 10^–4^)	0.5497657	0.5489796 (4.53 × 10^–3^)
4023	0.5443403	0.5443278 (6.93 × 10^–5^)	0.5494490	0.5489722 (2.73 × 10^–3^)
5896	0.5443370	0.5443244 (7.13 × 10^–5^)	0.5493035	0.5489508 (2.02 × 10^–3^)
lit.^c^	0.5443246			

a All densities in [bohr^–3^].

b Values of  in parentheses.

c The drachmanized value of  derived from an approximate electronic wave function involving 34020 explicitly correlated functions of the Hylleraas coordinates.^37^

**Table 7:** Spin Components of the One-Electron Densities of the Ground State of the Lithium Atom^a^

*K*	α electrons		β electrons	
	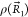	b	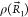	b
595	7.032914	7.037172 (3.11 × 10^–4^)	6.801711	6.805815 (3.09 × 10^–4^)
872	7.035053	7.037016 (1.43 × 10^–4^)	6.803765	6.805663 (1.43 × 10^–4^)
1278	7.035888	7.036964 (7.92 × 10^–5^)	6.804571	6.805617 (7.96 × 10^–5^)
1873	7.036156	7.036971 (5.97 × 10^–5^)	6.804888	6.805678 (5.98 × 10^–5^)
2745	7.036361	7.036965 (4.44 × 10^–5^)	6.805096	6.805681 (4.44 × 10^–5^)
4023	7.036715	7.036941 (1.66 × 10^–5^)	6.805463	6.805681 (1.66 × 10^–5^)
5896	7.036830	7.036938 (7.94 × 10^–6^)	6.805569	6.805674 (8.08 × 10^–6^)
lit.^c^	7.036930		6.805680	

a All densities in [bohr^–3^].

b Values of  in parentheses.

c Values derived from the respective total and spin one-electron densities listed in Table 8.

**Table 8:** Total and Spin One-Electron Densities of the Ground State of the Lithium Atom^a^

*K*	total		spin	
	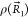	b	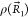	b
595	13.83462	13.84299 (3.10 × 10^–4^)	0.2312025	0.2313620 (3.94 × 10^–4^)
		[13.84299]		[0.2313573]
872	13.83882	13.84268 (1.43 × 10^–4^)	0.2312883	0.2313526 (1.43 × 10^–4^)
		[13.84268]		[0.2313526]
1278	13.84046	13.84258 (7.94 × 10^–5^)	0.2313168	0.2313483 (6.70 × 10^–5^)
		[13.84258]		[0.2313472]
1873	13.84104	13.84265 (5.97 × 10^–5^)	0.2312687	0.2312940 (5.13 × 10^–5^)
		[13.84265]		[0.2312944]
2745	13.84146	13.84265 (4.44 × 10^–5^)	0.2312647	0.2312844 (4.34 × 10^–5^)
		[13.84265]		[0.2312844]
4023	13.84218	13.84262 (1.66 × 10^–5^)	0.2312518	0.2312593 (1.66 × 10^–5^)
		[13.84262]		[0.2312593]
5896	13.84240	13.84261 (8.04 × 10^–6^)	0.2312616	0.2312644 (6.01 × 10^–6^)
		[13.84261]		[0.2312641]
lit.^c^	13.84261		0.2312497	

a All densities in [bohr^–3^].

b Values of  in parentheses, the sums and differences of the respective plain-capped spin components of the one-electron density (compiled in Table 7) in square brackets.

c The drachmanized values of the total and spin  derived from an approximate electronic wave function involving 34020 explicitly correlated functions of the Hylleraas coordinates.^37,38^

**Table 9:** On-Top Two-Electron Densities of the Ground State of the Beryllium Atom^a^

*K*	= 1.0 [bohr]		= 0.5 [bohr]	
	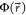	b	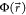	b
872	0.000180856	0.000178154 (9.52 × 10^–2^)	0.101918	0.101555 (1.29 × 10^–2^)
1278	0.000179659	0.000178048 (2.94 × 10^–2^)	0.101818	0.101565 (8.77 × 10^–3^)
1873	0.000179383	0.000178100 (3.23 × 10^–2^)	0.101706	0.101555 (4.83 × 10^–3^)
2745	0.000178711	0.000178022 (2.99 × 10^–2^)	0.101681	0.101575 (3.63 × 10^–3^)
4023	0.000178557	0.000178047 (1.01 × 10^–2^)	0.101640	0.101562 (3.80 × 10^–3^)
5896	0.000178345	0.000178043 (1.02 × 10^–2^)	0.101615	0.101563 (1.72 × 10^–3^)

*K*	= 0.2 [bohr]		= 0.1 [bohr]	
	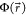	b	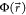	b
872	10.6789	10.6609 (5.06 × 10^–3^)	50.8515	50.7563 (5.92 × 10^–3^)
1278	10.6740	10.6614 (3.54 × 10^–3^)	50.8230	50.7568 (4.10 × 10^–3^)
1873	10.6682	10.6613 (1.97 × 10^–3^)	50.7929	50.7600 (1.97 × 10^–3^)
2745	10.6683	10.6633 (1.40 × 10^–3^)	50.7793	50.7536 (1.57 × 10^–3^)
4023	10.6654	10.6629 (7.23 × 10^–4^)	50.7712	50.7586 (7.62 × 10^–4^)
5896	10.6650	10.6627 (6.27 × 10^–4^)	50.7675	50.7562 (6.87 × 10^–4^)

*K*	= 0.05 [bohr]		= 0.02 [bohr]	
	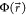	b	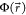	b
872	111.575	111.249 (1.77 × 10^–2^)	179.352	178.583 (3.58 × 10^–2^)
1278	111.466	111.241 (1.24 × 10^–2^)	179.186	178.588 (3.14 × 10^–2^)
1873	111.373	111.292 (2.48 × 10^–3^)	179.048	178.597 (3.50 × 10^–2^)
2745	111.325	111.243 (4.64 × 10^–3^)	178.876	178.642 (1.45 × 10^–2^)
4023	111.296	111.234 (4.24 × 10^–3^)	178.820	178.610 (3.35 × 10^–2^)
5896	111.289	111.241 (5.42 × 10^–3^)	178.778	178.653 (6.74 × 10^–3^)

a All densities in [bohr^–3^].

b Values of  in parentheses.

Several trends can be discerned in the data compiled in Tables 1–9. Examination of these data reveals that, overall, impressive accuracy improvements are attained with minimum computational effort. However, this improvement is uneven and in one case (the spin one-electron density at the nucleus of the lithium atom) there is actual worsening. The unevenness of the improvement stems from the fact that, unlike the EVI and IT approaches that sample the entire Cartesian space, the plain capping operates only in the vicinity of the two-particle coalescence points. The variations in the positions and spatial extents of the ECGs with *K* affect the fidelity with which the exact wave function is reproduced in these regions. As the lowering of the variational estimate of the ground-state energy upon the increase in the number of ECGs does not necessarily translate into a spatially even reduction of error in the wave function,^39^ the efficacy of plain capping varies widely with *K*. This unevenness is reflected in the radii of stitching that, while decreasing with *K* in almost all the cases studied, do not decay according to a simple power or exponential law. As already mentioned, the smallness of these radii obviates the need for the renormalization of the plain-capped densities.

The accuracy worsening observed upon the plain capping of the spin one-electron density at the nucleus of the lithium atom does not lend itself to obvious explanation. However, one should be reminded that increases of errors in the computed densities are also occasionally encountered in the case of the EVI-based approaches. For example, although the HSF formalism produces on average a 33-fold improvement in the accuracy of the one-electron densities at non-hydrogen nuclei,^40^ it yields grossly overestimated one-electron densities at hydrogens in molecules such as CH_3_^•^, H_2_O, and HF.^31,40,41^

## IV Conclusions

In summary, the plain capping constitutes a clever device for significantly improving accuracy of the one-electron, intracule, and on-top two-electron densities computed with cuspless basis functions. Unlike that effected by the previously proposed expectation value identities (EVIs), such as the Hiller-Sucher-Feinberg and Drachman formulas, and integral transforms, this improvement comes with negligible computational effort and minimum programming. Consequently, the plain capping is a viable alternative to those approaches, especially in the case of the on-top two-electron density, for which the respective EVIs are either nonexistent or unfeasible due to their excessively complicated implementation and prohibitive computational cost.
